# inFRank: a ranking-based identification of influential genes in biological networks

**DOI:** 10.18632/oncotarget.11878

**Published:** 2016-09-07

**Authors:** Xiuliang Cui, Xiaofeng Li, Jing Li, Xue Wang, Wen Sun, Zhuo Cheng, Jin Ding, Hongyang Wang

**Affiliations:** ^1^ The International Cooperation Laboratory on Signal Transduction, Shanghai 200433, China; ^2^ National Center for Liver Cancer, Eastern Hepatobiliary Surgery Institute, Shanghai 200433, China; ^3^ Department of Surgery, The Second Military Medical University, Shanghai 200433, China

**Keywords:** influential rank, network analysis, TCGA, liver hepatocellular carcinoma, prognosis

## Abstract

Capturing the predominant driver genes is critical in the analysis of high-throughput experimental data; however, existing methods scarcely include the unique characters of biological networks. Herein we introduced a ranking-based computational framework (inFRank) to rank the proteins by their influence. Using inFRank, we identified the top 20 influential genes in hepatocellular carcinoma (HCC). Network analysis revealed a prominent community composed of 7 influential genes. Intriguingly, five genes among the community were critical for mitotic spindle assembly checkpoint (SAC), suggesting that dysregulation of SAC could be a distinct feature of HCC and targeting SAC-associated genes might be a promising therapeutic strategy. Cox regression analysis revealed that CDC20 exerted as an independent risk factor for patient survival, indicating that CDC20 could be a novel biomarker for HCC prognosis. inFRank was then used for pan-cancer study, and all of the most influential genes in 18 cancers were achieved. We identified altogether 19 genes that were important in multiple cancers, and observed that cancers originating from the same organ or function-related organs tended to share more influential genes. Collectively, our results demonstrated that the inFRank was a powerful approach for deep interpretation of high-throughput data and better understanding of complex diseases.

## INTRODUCTION

Biomedical research is currently benefiting from the widespread use of high-throughput experimental technology, which provides us with valuable clues. However, because of the hundreds, and even thousands of differently expressed genes delivered outdistances the interpretation scope of most biomedical researchers, the data generated are not being fully utilized. The genes which usually affect numerous genes and are largely affected in the particular biological process are termed as “influential genes” and identification of the predominant influential genes in a specific cell process remains challenging. Methods initially designed to interpret complex networks have been used to settle this problem. One common strategy is to regard the genes with crucial topological properties such as hubs [1], and the genes in the center of network communities and modules [2, 3] as influential genes. Spreading process based algorithms [4] were also proposed to evaluate the influence of nodes, such as PageRank [5], LeaderRank [6] and physarumSpreader [7].

Distinct from other complex networks, biological networks possess several unique characteristics. First, the biological networks are hierarchically structured, and the network position of genes could make sense to their functions. Second, genes interact with each other in a dynamic manner, and it is necessary to select the “actually occurred” interactions for the given cellular context [8]. Third, most of the existing approaches consider only direct interactions; however, in most cases, genes could exert their influence on the expression of both direct and indirect downstream genes, especially during signaling transduction process. Fourth, despite the increasing availability of protein-protein interaction networks, such as HPRD [9], DIP [10], IntAct [11] and MINT [12], and BioGRID [13], considerable false-positives still exist [14].

Taking these concerns into consideration, in this study, we proposed a novel analysis pipeline referred to as influence rank (inFRank) to identify the most influential genes and sub-networks for a given biological phenomena. inFRank was used to achieve the influential genes in HCC, and was utilized in pan-cancer studies to discover the important genes for different cancers. Our results demonstrated that inFRank was a powerful method for deeply interpreting high-throughput experimental data and could provide better understanding of complex diseases, especially cancers.

## RESULTS

### Establishment of inFRank analysis method

To achieve the most influential genes from high-throughput experiment data, inFRank analysis method was established by three steps including background network construction, dynamic context network generation and influence score calculation as described below.

### Construction of background network

**Figure 1:**
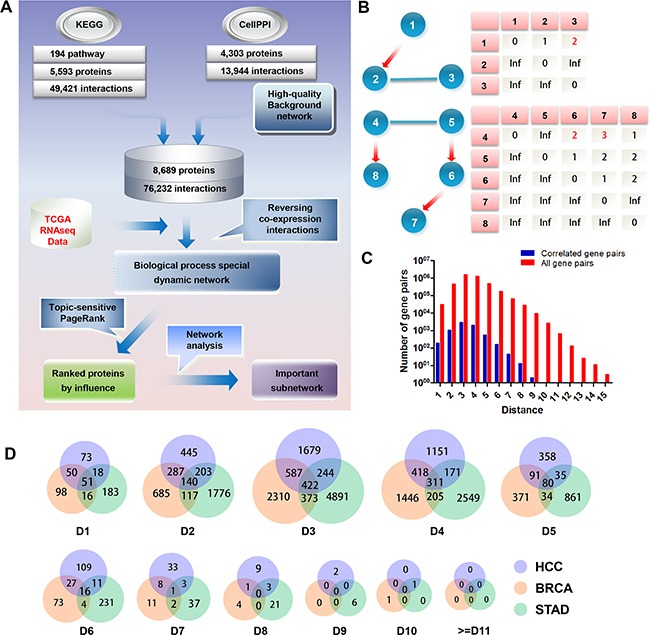
Establishment of inFRank analysis method **A.** The inFRank method was achieved in three steps. First, we constructed a high-quality static background network by integrating KEGG pathways and protein-protein interaction networks. Second, we used gene expression information and generated a special biological process dynamic network by filtering co-expressed interactions. Finally, the topic-sensitive PageRank algorithm was used to calculate the influence score for each protein and rank them by their influence scores. **B.** Schematic diagram of network distance definition. The red directed edges represent interactions from KEGG pathways, and the blue undirected edges represent interactions from protein-protein interaction networks. The network distances are listed in the table below. **C.** The ratio of correlated gene pairs to all of the gene pairs in the HCC network. **D.** The Venn diagram of the co-expressed gene pairs of HCC, BRCA and STAD. Ds stand for Distance.

By integrating KEGG pathways and the protein-protein interaction network from the work by Rolland *et al* [15], we constructed the high-quality background network consisting of 77,232 interactions between 8,689 proteins. The network distance was calculated, and the distance was defined as the minimal number of consecutive steps required to arrive from one protein to another on the background network. The max distance in our background network is 15. As our background network contains both directed and undirected interactions, we redefined the upstream and downstream relationship between genes. As illustrated in Figure 1B, when there was binding relationship between node 3 and node 2, which is the direct downstream of node 1, we treated node 3 as the downstream of node 1, and defined the distance from node 1 to node 3 as 2. Similarly, we treated node 4 as the upstream of node 6, and defined the distance from node 4 to node 6 as 2.

### Generation of biological process specific dynamic network

We weighted the background network using gene expression data, and reserved significantly co-expressed gene pairs (the absolute value of pearson correlation coefficient (PCC) of their expression is more than 0.7 [16, 17]) ground on the suppose that truly interacting gene pairs tend to have a correlated expression pattern. The influence of the upstream gene was assumed to become weaker as the network distance increased. Therefore, the final weight of the interaction was defined as the quotient of PCC and the network distance between the protein pairs.

We used the RNA-seq data of HCC to illustrate how the gene pairs were selected. There were 31,335 directly interacting gene pairs, of which only 192 were co-expressed. The ratio of co-expressed gene pairs to all gene pairs with a different network distance is shown in Figure 1C. These data indicated that only a minor proportion of interactions were associated with HCC development, and our results also emphasized the necessity of constructing a cellular process specific dynamic network.

Furthermore, we investigated whether the co-expressed gene pairs in HCC were also co-expressed in other cancers. The relationship of co-expressed gene pairs with different network distance in HCC, Breast invasive carcinoma (BRCA) and Stomach adenocarcinoma (STAD) is shown in Figure 1D. We found that gene pairs co-expressed in one cancer were not necessarily co-expressed in other cancers, and only a minority fraction of interactions involved in different cancers.

### Identification of influential genes in HCC identified by inFRank

#### The influence rank converged rapidly with the increase of network distance

**Figure 2:**
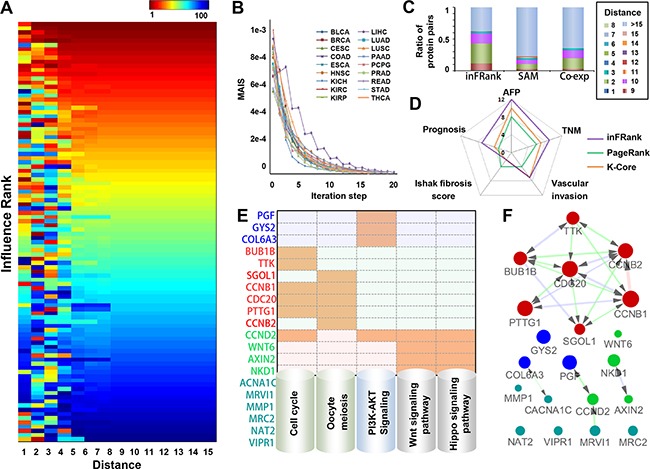
Identification of influential genes in HCC by inFRank **A.** The heatmap of the top 100 influential genes by rank using a different value as the network distance cutoff. **B.** The convergence of inFRank in 18 cancer datasets. **C.** The distribution of network distance between the top 100 genes identified by three different methods. **D.** The number of genes associated with AFP, TNM staging, vascular invasion, Ishak fibrosis score and prognosis in the most influential genes identified by inFRank, k-core and PageRank. **E.** The KEGG pathway analysis of the 20 most influential genes identified by inFRank. Five pathways were detected, which include four or more genes in the top 20 list. An orange mark indicates that the gene is in the corresponding pathway. **F**. The sub-network of the 20 most influential genes. The red edge represented direct interactions (network distance 1), green edges linked gene pairs with a network distance of 2, and blue edges linked gene pairs with network distance of 3.

We first used inFRank to achieve influential genes in HCC using the RNA-seq data of 320 HCC samples and 50 normal controls from TCGA. To investigate the impact of network distance to the convergence of the rank result, we simulated the ranking procedure using different network distance cutoffs. We first reserved only gene pairs with distance 1 (directly interacted pairs), and calculated the influence rank (*IR*_1_) for genes in the network. Then, we reserved gene pairs with distance less than 2 (2 was included), and calculated the influence rank *IR*_2_ as the same. As the max distance in the background network is 15, the procedure was repeated 15 times until we got *IR*_15_, and *IR*_15_ was used as the final influence score. We ranked the genes according to *IR*_15_, and selected 100 genes with top ranks. We compared the ranks of these 100 genes in all the 15 rank lists. As shown in Figure 2A, we found that the ranks converged rapidly with the increment of network distance. The Spearman's rank correlation coefficient (ρ) between the relative ranks of *IR*_1_-*IR*_14_ and *IR*_15_ of 100 genes ρ(*IR*_1_…*IR*_14_…*IR*_15_) (Supplementary Figure S1), and the ρ also increased rapidly with the increment of network distance. We noticed that ρ(*IR*_4_, *IR*_15_) already reached 0.85, which indicated that gene pairs with network distance more than 4 could have limited impact on each other.

The convergence of inFRank was investigated using 18 cancer RNA-seq datasets. It was considered converged when MAIS was less than 1.0e-5. As shown in Figure 2B, all the 18 cases were converged and the iteration step was from 14 to 20. To evaluate the influence of sample size to the results of inFRank, we randomly selected 160 HCC samples from the total amount of 320 samples for 1000 times and calculated the influence scores respectively. The number of overlaped genes between the top 20 influential genes identified using randomly selected 160 samples and that identified using total 320 samples is 18.91±0.62, which indicates that the influence of sample size of RNA-Seq data input on the result of inFRank predication is minor.

#### Comparison of influential genes identified by inFRank and other methods

The genes involved in a cellular process tend to interact with each other, and the isolated genes are more likely to be noise. To illustrate the robustness of inFRank to the noise of high-throughput data, we compared the network distance between the 100 most important genes identified by the following 3 methods: 1) 100 most differentially expressed genes by SAM method [18] with the R package “samr”; 2) 100 co-expressed genes with highest PCC scores; 3) the 100 most influential genes by inFRank. As shown in Figure 2C, inFRank outperformed the other two methods, as evidenced by the much fewer isolated genes identified by inFRank, and the 100 most influential genes identified by inFRank were closer than the other two methods.

To further demonstrate the efficiency of our method, we compared the function of the 20 most influential genes identified by inFRank and other two network-based methods. The first set contained 20 significantly different expressed genes with the highest coreness values, the second set was 20 influential genes identified by PageRank, and the third set include 20 genes with highest influence score identified by inFRank. As shown in Figure 2D, there were more genes associated with AFP, TNM staging, vascular invasion and prognosis in the third gene set, detailed information were in Supplementary Table S3-S5. These results indicated that our method was more efficient in identifying function important genes. The 20 genes identified by inFRank were mainly involved in 5 pathways including cell cycle, oocyte meiosis, PI3K-Akt signaling pathway, Wnt signaling pathway and Hippo signaling pathway (Figure 2E). These pathways are all acknowledged to play important roles in cancers particularly in HCC. In further study, these 20 genes were used as seed genes to construct the activated sub-network (Figure 2F). A key network module was achieved, which consisted of 7 densely connected genes including CDC20, TTK, BUB1B, PTTG1, SGOL1, CCNB1 and CCNB2. Gene ontology illustrated that most of them exert important functions throughout G2/M phase of the cell cycle.

#### inFRank-identified genes exhibited superior clinical significance

**Figure 3:**
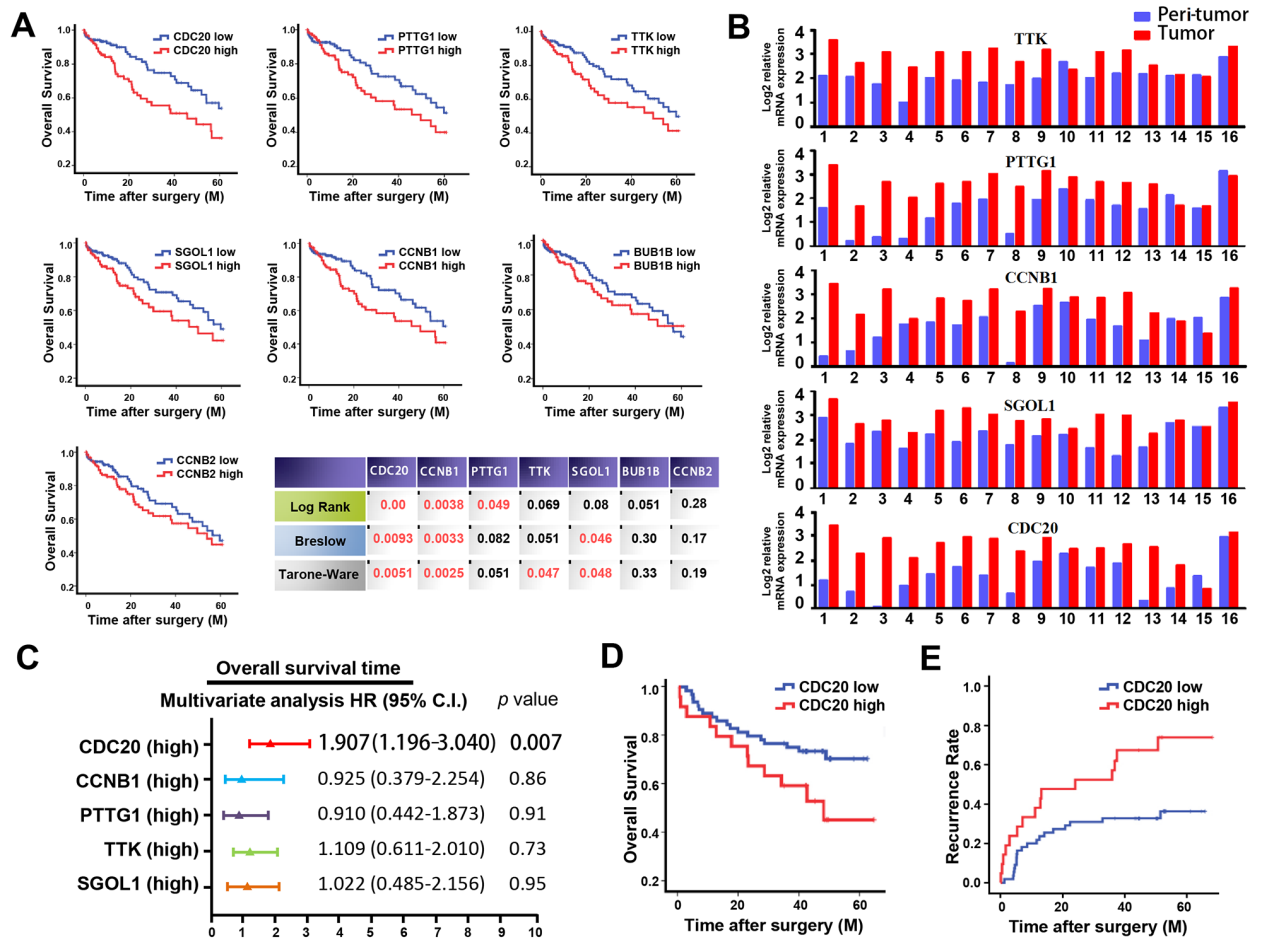
inFRank-identified genes exhibit superior clinical significance in HCC patients **A.** Overall survival rates of 320 HCC patients with high or low expression of 7 genes using 3 different analysis method. **B.** Comparison of the expression levels of 5 genes in 16 HCC tumor tissues and peri-tumor normal tissues. **C.** Multivariate analysis of hazard ratios for the overall survival time of the 320 patients. **D.** Overexpression of CDC20 correlated with the poor survival in our cohort of 76 HCC patients. **E.** Overexpression of CDC20 correlated with the early recurrence in our cohort.

Expression of the 7 genes identified was remarkably increased in HCCs compared with para-cancerous normal tissues (Supplementary Figure S2). We then investigated the correlation between the expression of these genes and patient prognosis. As shown in Figure 3A, HCC patients with highly expressed CDC20, PTTG1, TTK, SGOL1 or CCNB1 exhibited shorter survival time than the patients with low expression of these genes. Interestingly, all of the five genes were spindle assembly checkpoint (SAC)-associated genes. The SAC is a ubiquitous safety device that maintains genomic stability through delaying chromosome segregation until all the chromosomes have attached to the mitotic spindle [19]. SAC defect is closely associated with genome instability including gene mutation, amplification or chromosomal aberrations. Collectively, these data suggested that dysregulation of SAC might be a distinct feature of HCC.

To further confirm the up-regulation of these 5 genes in HCC, realtime PCR was conducted using 16 paired human HCC specimens. As expected, the expression levels of the 5 genes were all higher in HCC samples compared to the adjacent normal controls (Figure 3B). In further study, relationship between the expression of these 5 genes and clinic-pathological factors of patients was analyzed. As shown in Table 1, overexpression of these genes in HCC was significantly associated with tumor grade, stage and metastasis, suggesting that the influential genes identified by inFRank could play important roles in HCC development. Consistently, overexpression of PTTG1 [20], TTK [21], SGOL1 [22] and CCNB1 [23] have been reported correlate with poor survival of HCC patients, while the significance of CDC20 in HCC prognosis remains unknown.

**Table 1:** Correlation between influential genes expression and Clinico-pathological characteristics of HCC

Variable	CDC20			PTTG1			TTK			SGOL1			CCNB1		
	Low	High	p	Low	High	p	Low	High	p	Low	High	p	Low	High	p
Age															
>60y	95	80	0.12	95	80	0.12	104	71	0.0003	99	76	0.013	95	80	0.12
Gender															
Male	111	102	0.34	110	103	0.48	110	103	0.48	114	99	0.097	112	101	0.24
Female	49	58		50	57		50	57		46	61		48	59	
Etiology															
Hepatitis B	89	75	0.15	88	76	0.22	83	81	0.91	83	81	0.91	77	87	0.31
Hepatitis C	44	31	0.11	44	31	0.19	43	32	0.19	42	33	0.29	29	46	0.034
Alcohol	65	55	0.30	61	59	0.91	73	47	0.0038	69	51	0.049	64	56	0.42
Tumor grade			0.0037			0.0004			9.5e-6			1.7e-5			5.5e-5
G1	32	15		32	15		34	13		32	15		35	12	
G2	83	73		86	70		87	69		89	67		83	73	
G3	42	67		40	69		37	72		38	71		39	70	
G4	3	5		2	6		2	6		1	7		3	5	
Ajcc pathologic tumor stage			3.0e-5			3.2e-6			1.2e-5			7.6e-5			8.1e-8
Stage I	98	59		101	56		99	58		98	59		103	54	
Stage II	30	48		30	48		29	49		32	46		28	50	
Stage III	28	51		26	53		28	51		27	52		25	54	
Stage IV	4	1		3	2		4	1		3	2		4	1	
Vascular invasion			0.425			0.15			0.24			0.57			0.015
Micro	39	42		38	43		36	45		41	40		35	46	
Macro	4	8		3	9		4	8		4	8		2	10	
None	117	110		119	108		120	107		115	112		123	104	

#### CDC20 was identified as a novel biomarker for patient prognosis

CDC20 is one of the co-activators of anaphase promoting complex (APC). Mounting evidence has implied that CDC20 exerts oncogenic function in human tumorigenesis including pancreatic cancer [24], breast cancer [25], colorectal cancer [26], and lung cancer [27]. Enhanced CDC20 expression was reported to be involved in HCC development, but its clinical significance in patient prognosis remains obscure [28]. As shown in Figure 3C, multivariate analysis revealed that CDC20 is the only independent prognostic factor for patient survival among the 5 genes achieved (hazard ratio 1.907, 95% CI 1.196-3.040, *p*=0.007). Moreover, significance of CDC20 in HCC prognosis was validated by realtime PCR data from our cohort of 76 HCC patients, and the patients in the CDC20 high group exhibited shorter survival time than those patients in CDC20 low group (median OS time were 37 and 45 months, respectively; difference=8 months; *p*<0.05) as shown in Figure 3D. Furthermore, we investigated the correlation of CDC20 expression with HCC recurrence. As shown in Figure 3E, expression level of CDC20 in patients correlated significantly with HCC recurrence (*p*=0.003). Collectively, these data suggested that CDC20 could serve as a novel biomarker for HCC prognosis and recurrence.

#### CDC20 played an essential role in HCC cell proliferation and invasion

**Figure 4:**
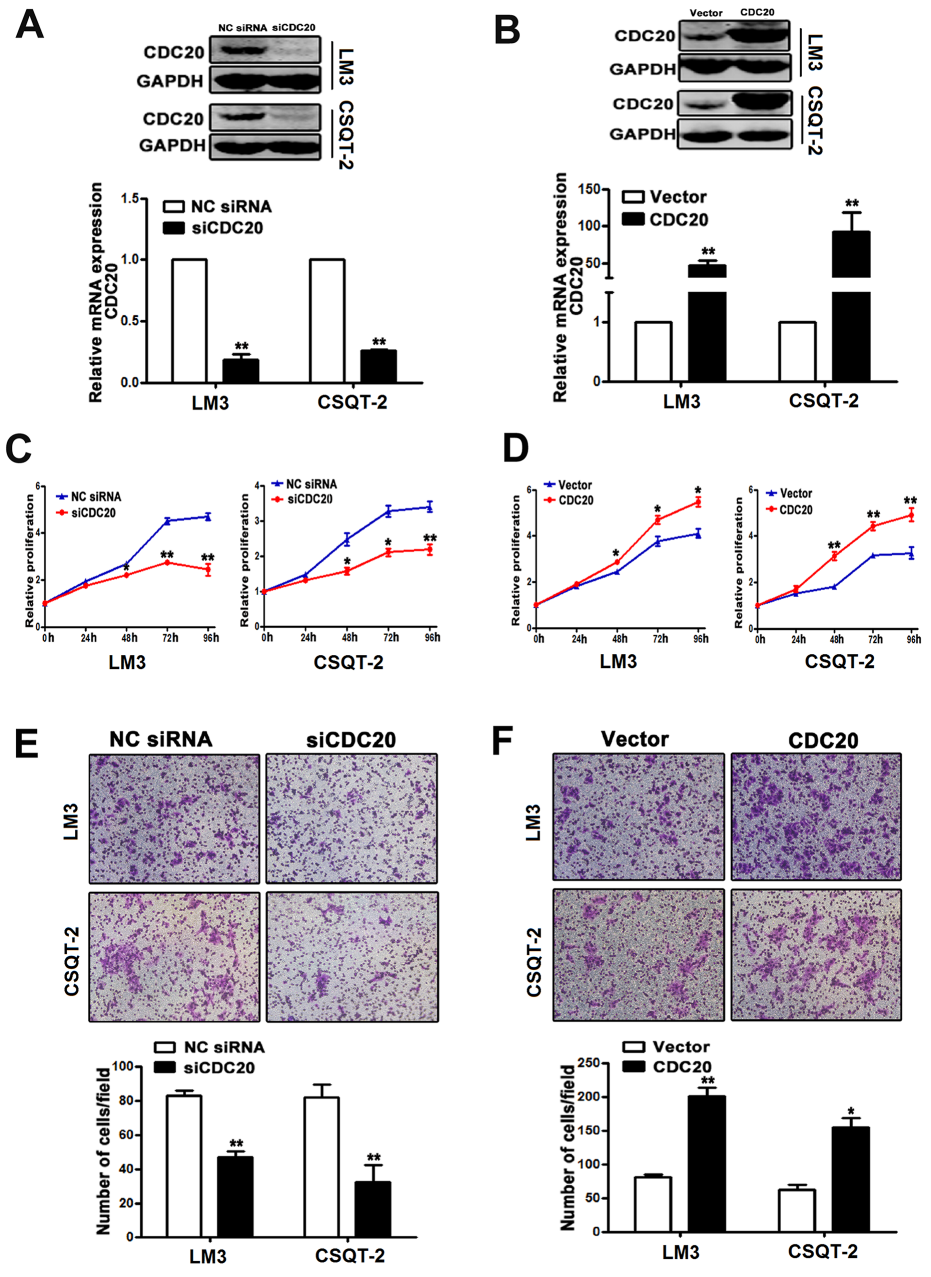
Effect of CDC20 knockdown and overexpression on HCC cell behavior **A**. The mRNA and protein levels of CDC20 were notably reduced by CDC20 siRNA transfection in LM3 and CSQT-2 cells. **B**. Overexpression of CDC20 was identified in LM3 and CSQT-2 cells. **C**. LM3 and CSQT-2 cells transfected with siCDC20 exhibited significantly reduced proliferation compared with the cells transfected with NC siRNA. **D**. LM3 and CSQT-2 cells transfected with pCMV3/CDC20 plasmid showed remarkably enhanced proliferation compared with the cells transfected with vector control. **E**. The invasive property of LM3 and CSQT-2 cells were decreased by CDC20 knockdown. **F**. CDC20 overexpression increased the invasive property of LM3 and CSQT-2 cells *, p<0.05, **, P<0.01.

Considering the functional relevance of CDC20 in HCC remains unclear, we thereafter investigated the role of CDC20 in HCC cells using overexpression and knockdown approaches. Knockdown efficiency of siCDC20 and overexpression of CDC20 was validated by realtime PCR and western blot in LM3 and CSQT-2 cells respectively (Figure 4A&4B). As shown in Figure 4C&4D, interference of CDC20 significantly decreased the proliferation of HCC cells, and CDC20 overexpression potently increased the HCC cell proliferation. Furthermore, cell invasion assay demonstrated that knockdown of CDC20 impaired the invasion ability of HCC cells (Figure 4E) and forced CDC20 expression enhanced HCC cell invasion (Figure 4F). Taken together, these data suggested that CDC20 could play an important role in HCC progression.

### Pan-cancer analysis using inFRank-identified influential genes

**Figure 5:**
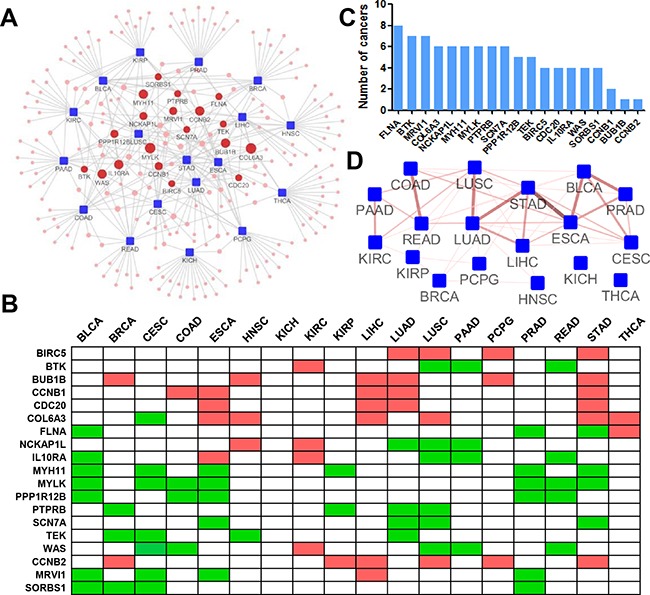
Pan-cancer analysis using inFRank-identified influential genes **A.** The network of the 20 most influential genes and cancers. The cancers (blue rectangle nodes) are linked to their 20 most influential genes (red round nodes) by a grey line. The size of the gene nodes corresponds to the number of directly connected cancers. **B.** Heatmap of 19 genes for 18 cancers. A list of the top 20 influential genes for more than 4 (4 were included) types of cancers. A green mark indicates the gene is among the top 20 influential genes and is down regulated in tumor samples, and a red mark denotes up regulated genes. **C.** The number of cancers whose prognosis were associated with the expression of 19 genes. **D.** The connective map of 18 cancers. Two cancers (blue rectangle nodes) are linked if there is an overlap between their top 20 influential genes. The color and thickness of the edge correspond to the number of shared genes.

We employed inFRank to calculate the gene influence scores for 18 cancers and identified the 20 most influential genes for each cancer (Figure 5A & Supplementary Table S3). There were 229 unique genes, 165 of which appeared to have considerable influence for only one cancer, suggesting the distinct regulatory network in different cancer types. The distribution was shown in Supplementary Figure S3. Interestingly, 19 genes were identified to be influential in multiple cancers, implying their universal role in carcinogenesis (Figure 5B). We thereafter investigated the correlation between the expression of these genes and the patient prognosis. In average, each of the 19 genes correlates with the prognosis of 4.84±1.95 cancer types, which was significantly higher than the number of cancer types (2.11±1.72, *p*=1.5e-7) from randomly selected genes (Figure 5C). In addition, we investigated the relationship between different cancers based on the overlap analysis of the most influential genes of each cancer. The association network is shown in Figure 5D. We observed that distinct cancers originating from the same or closely related organ or tissues tended to share influential genes. There were 4 pairs of cancers including lung adenocarcinoma (LUAD)and lung squamous cell carcinoma (LUSC), esophageal carcinoma (ESCA) and stomach adenocarcinoma (STAD), rectum adenocarcinoma (READ) and colon adenocarcinoma (COAD), and bladder urothelial carcinoma (BLCA) and prostate adenocarcinoma (PRAD) shared 6 influential genes, respectively, suggesting the significance of inFRank in dissecting the complex diseases.

## DISCUSSION

To capture the predominant driver genes for the given cellular process in analysis of high-throughput experimental data remains a great challenge due to the large-scale of the data and the redundancy of the information. Network-based methods have been anticipated to settle these problems. However, current approaches usually fail to include the unique characters of biomedical networks. Herein we developed inFRank, a ranking-based method for identifying influential genes, which particularly emphasizes the four features of biomedical networks. First, the biological networks are hierarchical, and the abnormal expression of upstream regulatory factors will have a significant impact on the downstream genes; however, the differential expression of downstream regulatory factors will have a relatively little impact on upstream factors [29]. Second, current biological networks provide us with the all possible relationships between proteins; however, proteins exert different biological functions by interacting with distinct partners dynamically. Therefore, it is necessary to select the true interactions for the given cellular context [8]. Third, most of the traditional approaches rely on the direct interactions only; however, in some cases a biological effect could be reflected by the expression alteration of the indirect downstream factors. Therefore, the upstream regulatory relationship in our algorithm is not limited to directly interacting gene pairs, but also includes indirect regulatory relationships, making it possible to discover new interactions. The last but not the least, there are a considerable number of false-positives in the current protein-protein interaction networks [14], which requires a filtering process to reduce the effect of false-positives.

We used inFRank to calculate the influence score of genes expressed in HCC, and found the influential genes identified tend to have smaller network distance, which indicated that inFRank was robust to the noise of high-throughput experimental data. To further validate the efficiency of our algorithm, we compared our method with k-core and PageRank algorithm, and the influential genes identified by inFRank were more likely to be associated with AFP, TNM stage, vascular invasion and prognosis. Together, these findings further supported that inFRank analysis which was designed ground on the principle of biomedical networks, could achieve function important molecules for the given cellular process effectively.

In present study, we found that 5 of 7 influential genes in the key network community were SAC-associated genes, suggesting that dysregulation of SAC could be a distinct feature of HCC. Moreover, targeting SAC-associated genes could be a promising therapeutic strategy in HCC treatment. In addition, multivariate analysis showed that CDC20 exerted as an independent prognostic factor for patient survival, which was validated by our independent patient cohort. These results demonstrated the efficiency of inFRank in identifying influential genes in complex diseases including cancers.

Although different cancers exhibit distinct pathogenesis, there are certain hallmarks shared in various cancer types [30]. Pan-cancer studies have revealed the intra-cancer heterogeneities and cross-cancer similarities [31–33]. In present study, inFRank was used for pan-cancer investigation based on TCGA data set, and the influential genes in 18 cancer types were identified. We found that cancers originating from functional related organs or tissues are prone to share influential genes, which is worthy of further investigation. In addition to RNA-seq data, inFRank could also be applied in analyzing other types of omics data, such as proteomic and metabolic data. Moreover, inFRank method could also be applied to interpret RNA-Seq dataset of other biological processes or disease such as chronic inflammation, cardiovascular diseases and diabetics *etc*.

## MATERIALS AND METHODS

### RNA-seq data from 18 cancers

The Illumina HiSeq mRNA expression data of 18 cancers were downloaded from the TCGA database (http://cancergenome.nih.gov/). There were 6,624 tumor samples and 651 normal samples in our analysis (detailed information is in the Supplementary Table S1). Level 3 normalized values by RNA-Seq by Expectation Maximization (RSEM) algorithm were used as the expression levels for the corresponding genes. We removed those genes whose expression were absent in more than 10% of samples before analysis.

### Background biological networks

The manually drawn KEGG pathway maps provide us with precious knowledge on the molecular interactions [34]. We downloaded the .xml files for 194 non-metabolic pathways from KEGG, and parsed them into directed networks using Perl script. Then, these separate pathways were merged into a global KEGG network, which contained 49,421 interactions between 5,593 proteins. To avoid the noise due to the false-positives of several current protein-protein interaction networks, we used a previously reported data set [15], which is a systematic map of 13,944 high-quality binary interactions between 4,303 proteins.

### Patient cohort and HCC samples for validation

A total of 94 HCC tissues were obtained from the Eastern Hepatobiliary Surgery Hospital (Shanghai, China). Sixteen patient HCC samples with paired peri-tumor normal tissues were used to validate the expression of genes of interest. Seventy eight HCC tissue samples with clinico-pathological information were randomly retrieved from HCC patients who underwent curative resection at the Eastern Hepatobiliary Surgery Hospital from March 2009 to September 2010. All patients were followed up until February 2015, with a median observation time of 44.6 months. Overall survival (OS) was defined as the interval between the dates of surgery and death. Disease-free survival (DFS) was defined as the interval between the dates of surgery and recurrence; if recurrence was not diagnosed, patients were censored on the date of death or the last follow-up. Overall survival analysis was performed using the Kaplan-Meier method [35]. The procedure of human sample collection was approved by the Ethics Committee of the Eastern Hepatobiliary Surgery Hospital.

### Topic-sensitive PageRank

The PageRank algorithm was first proposed by Brin and Page [36], which was originally used to evaluate webpages by producing an authority score to show the importance of each webpage. This algorithm was also used to identify important genes recently [2, 37]. However, the authority score produced by the original PageRank algorithm rely only on the topological structure of biological networks. Topic-sensitive PageRank (TSPR) algorithm confers high weight on pages strongly associated with the query term and generates more accurate query-specific ranking scores by including topic-sensitive rank vectors [38]. Similarly, genes with high expression fold change (FC) are more likely to participate in the carcinogenesis process, and deserve high influential score. In inFRank, the FC of the gene was used as the topic-sensitive vector, which enabled us avoid topological bias and integrate gene expression information to evaluate the influence of genes.

TSPR algorithm was performed in an iterative manner, and the influence score was used as the input of the next iteration. Let N equal the number of nodes in the network, the initial score ν_*i*_(0) for each node *i* is set to be 1/N. The influence score ν_*i*_(t) for node *i* at time step *t* is calculated as:

$$v_{i}(t)\;=\;(1\;-\;\beta )\;Mv_{i}(t-1)\;+\;s\frac{\beta }{\left|s\right|}$$ (1)

where *M* is the transition matrix of the dynamic network, ν_*i*_(*t*-1) is the score for node *i* at time step *t*-1. *s* is the FC vector and *β* is 0.2 in our experiments as conventionally.

The difference of influence score Δν_*i*_(*t*) for node *i* at time step *t* is calculated as formula 2, and iteration process is stopped until the maximum absolute value of influence scores (MAIS) for all nodes achieves less than 1.0e-5.

$$△v_{i}(t)\;=\;v_{i}(t)\;-\;v_{i}(t-1)$$ (2)

The significance was estimated based on the distribution of the influence scores computed by a random simulation procedure. We constructed a random network, and assigned the fold change values to genes randomly. The process was repeated 1000 times, and for each time, the gene influential score was calculated by inFRank. Let n be the number of influential scores from the distribution that are greater than the actual value for the gene. The estimate of the p value was computed as *p* = (*n*+1)/1000.

### Real-time PCR assay

The original amount of the specific transcripts was measured using quantitative real-time PCR (qRT-PCR) with a SYBR Green PCR Kit (Applied Biosystems, Foster City, CA) and a LightCycler 96 Real-Time PCR System (Roche, Mannheim, Germany). The mRNA levels of cell division cycle 20 (CDC20), TTK Protein Kinase (TTK), pituitary tumor-transforming protein 1 (PTTG1), shugoshin-like protein 1 (SGOL1) and cyclin B1 (CCNB1) in HCCs were normalized against β-actin. The primer sequences were provided in Supplementary Table S2.

### Cell transfection and western blot analysis

HCC cell lines HCC-LM3 (LM3) and CSQT-2 which was established from patient portal vein tumor thrombus (PVTT) were cultured in DMEM supplemented with 10% FBS (Gibco, Invitrogen). Double-stranded, small interfering RNA (siRNA) was synthesized and purified by GenePharma (Shanghai, China). Sense, 5′-GCAGAAACGGCUUCGAAAUTT-3′; antisense, 5′-AUUUCGAAGCCGUUUCUGCTT-3′. A scrambled non-targeting siRNA was used as negative control. pCMV3/CDC20 plasmid and the vector control were purchased from Sino Biological (Beijing, China). The HCC cells were transfected with pCMV3/CDC20 or small interference RNA of CDC20 and their control respectively using the lipo2000 reagent as described previously [39]. Western blotting assay was performed to validate the overexpression or knockdown efficiency of CDC20 in HCC cells using anti-CDC20 antibody (Cell Signaling Technology Inc, Danvers, MA). The blots were normalized with GAPDH.

### Cell proliferation and invasion assay

The HCC cells transfected with pCMV3/CDC20 or siCDC20 as well as the corresponding controls were seeded into 96-well plate (3000 cells/well). The cell proliferation rates were compared using Cell Counting Kit-8 (Dojindo Laboratories, Japan) at distinct time points as described previously [39]. For invasion assay, 1×10^5^ cells were added to the upper chamber of a polycarbonate transwell filter coated with 30 mg/cm^2^ Matrigel (Sigma-Aldrich, St.Louis, MO). After 24 hours at 37°C, cells on the lower surface of the membrane were fixed by 4% formaldehyde for 30 minutes and stained, photographed and counted under a microscope as described previously [39].

### Statistical analysis

Differences among the variables were assessed by a c^2^ analysis or two-tailed Student *t* test. Kaplan-Meier analysis and log-rank test was used to assess patient survival between subgroups. Multivariate analysis was performed by a cox proportional hazards model analysis.

## SUPPLEMENTARY MATERIALS FIGURES AND TABLES


