# Supplementary material for: inFRank: a ranking-based identification of influential genes in biological networks
Source: Oncotarget. 2016 Sep 7;8(27):43810–21. doi: 10.18632/oncotarget.11878 (PMC5546442; doi:10.18632/oncotarget.11878)
Supplement: Supplementary file 1 [file oncotarget-08-43810-s001.pdf]

inFRank: a ranking-based identification of influential genes in biological networks

SUPPLEMENTARY FIGURES AND TABLES

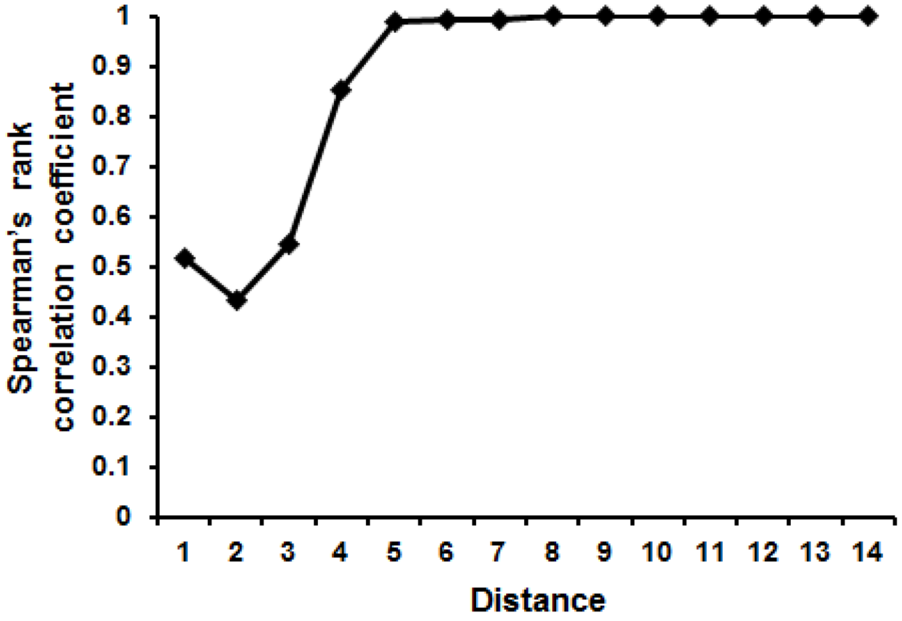

Supplementary Figure S1: The Spearman's rank correlation coefficient (ρ) between the relative ranks of IR_1-IR_{14} and IR_{15} of 100 genes $\rho(IR_1 \ldots IR_{14}, IR_{15})$.

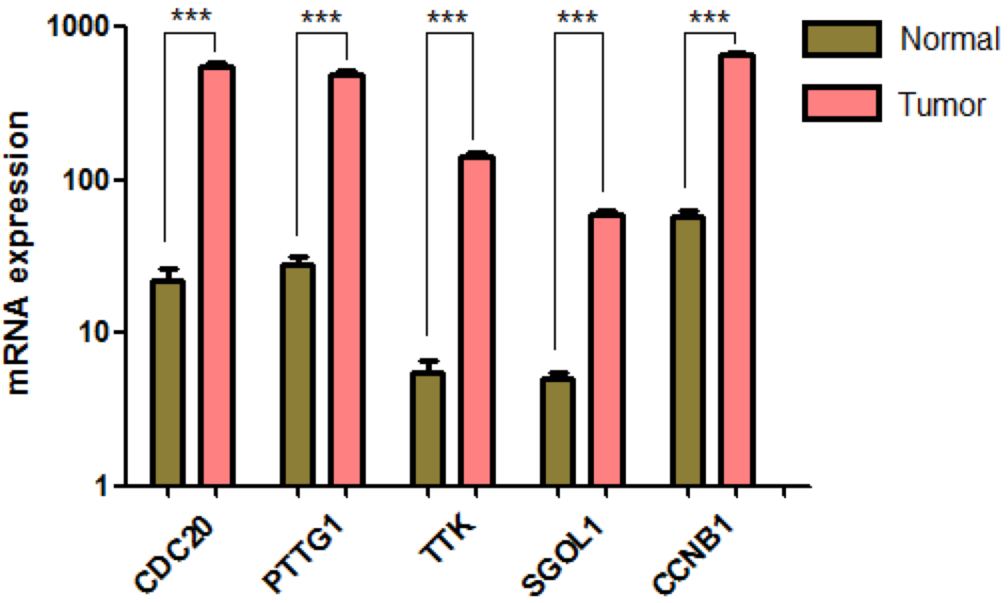

Supplementary Figure S2: Expression of the 5 genes identified was remarkably increased in HCCs compared with para-cancerous normal tissues.

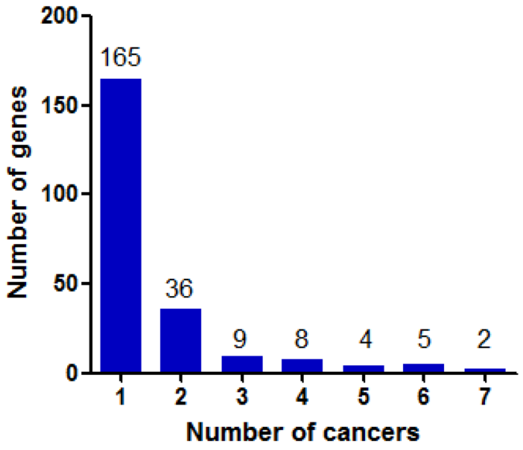

Supplementary Figure S3: The distribution of 229 influential genes in cancers.

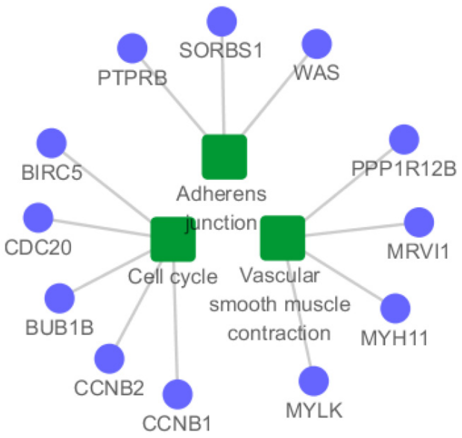

Supplementary Figure S4: The pathway enrichment analysis of influential genes identified by inFRank. The green rectangle nodes represented enriched pathways, and blue nodes represented genes. The nodes are linked if the gene was involved in the pathway.

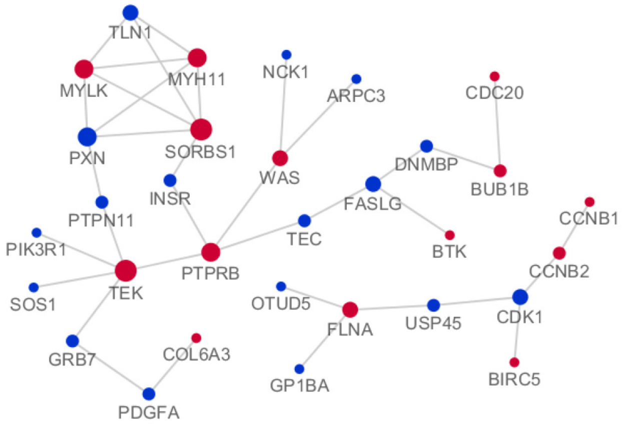

Supplementary Figure S5: The sub-network of 19 genes. The red nodes represented seed genes, and blue nodes represented genes with interacting relationship with the seed genes. The size of each node corresponds to their interacting neighbors.

Supplementary Table S1: Information of TCGA samples used in our analysis

TCGA acronym	Cancer type	Number of tumor samples	Number of normal samples
**BLCA**	Bladder urothelial carcinoma	391	19
**BRCA**	Breast invasive carcinoma	1049	112
**CESC**	Cervical squamous cell carcinoma and endocervical adenocarcinoma	302	3
**COAD**	Colon adenocarcinoma	278	26
**ESCA**	Esophageal carcinoma	173	13
**HNSC**	Head/neck squamous cell carcinoma	511	42
**KICH**	Kidney chromophobe	91	25
**KIRC**	Kidney renal clear cell carcinoma	512	72
**KIRP**	Kidney renal papillary cell carcinoma	273	32
**LIHC**	Liver hepatocellular carcinoma	320	50
**LUAD**	Lung adenocarcinoma	509	57
**LUSC**	Lung squamous cell carcinoma	424	43
**PAAD**	Pancreatic adenocarcinoma	168	4
**PCPG**	Pheochromocytoma and paraganglioma	179	3
**PRAD**	Prostate adenocarcinoma	484	52
**READ**	Rectum adenocarcinoma	94	6
**STAD**	Stomach adenocarcinoma	363	33
**THCA**	Thyroid carcinoma	503	59

Supplementary Table S2: Primers of Real-time PCR

Gene		Primer sequence
**β-actin(Human)**	Forward (5′- 3′)	GGCCCAGAATGCAGTTCGCCTT
	Reverse (5′- 3′)	AATGGCACCCTGCTCACGCA
**CDC20 (Human)**	Forward (5′- 3′)	CCCTGCCAGACCGTATCC
	Reverse (5′- 3′)	CAGCCAAGTAGTTGCCCTC
**PTTG1 (Human)**	Forward (5′- 3′)	GGGTCTGGACCTTCAATCAA
	Reverse (5′- 3′)	GGCAGGAACAGAGCTTTTTG
**CCNB1 (Human)**	Forward (5′- 3′)	CGGGAAGTCACTGGAAACAT
	Reverse (5′- 3′)	AAACATGGCAGTGACACCAA
**TTK (Human)**	Forward (5′- 3′)	TCAAGGAACCTCTGGTGTCA
	Reverse (5′- 3′)	GGTTACTCTCTGGAACCTCTGGT
**SGOL1 (Human)**	Forward (5′- 3′)	AGG CAA AAGAT GGCC AA GGA
	Reverse (5′- 3′)	AAA GAC CTG CGT TTGCCA AT

Supplementary Table S3: The 20 most influential genes of 18 cancers

Cancer	Influential genes
**BLCA**	MYLK, PPP1R12B, GZMB, FLNA, CACNA2D3, ATP1A2, MRVI1, IL10RA, MYH11, CFL2, SORBS1, SGCD, PSD, CACNA1C, MAOB, PRKG1, NKD1, RAB23, PDE1C, DMD
**BRCA**	PDE2A, PPARG, FZD4, ITGA7, BUB1B, TEK, PPP1R1A, SORBS1, GNAI1, GNG11, PRKAR2B, CEBPA, CCNB2, CD36, TNNC1, PTPRB, GNG2, ACACB, EPAS1, ORC1L
**CESC**	COL6A3, CXCL12, CACNB2, MYH11, CACNA1C, JAM2, KLRC2, MRVI1, FIGF, MYLK, PTGER3, TEK, CACNA1G, KCNMB1, SORBS1, DES, COL1A2, CCL14, HLA-DMA, WAS
**COAD**	CCNB1, CA2, CDKN2B, MPDZ, GYPC, CACNA2D1, PTTG1, MYLK, UGT1A10, PHLPP2, PLCE1, ITGA7, CHP2, LIFR, PDE2A, PPP1R12B, WAS, PSD, JAM2, JAM3
**ESCA**	HNF4A, CDC20, MYLK, ITPR1, COL6A3, CCNB1, HKDC1, SCN7A, MRVI1, ITGA7, SGCD, CYP17A1, ADCY5, COL2A1, TACR2, MYH11, IL10RA, PPP1R12B, SNAI2, DUSP7
**HNSC**	PDGFRB, WNT6, COL6A3, FGFR4, HLA-C, ATP6V1B1, TNNC2, TTN, CFL2, OAS2, TEK, ACTN2, TNNC1, SLC25A4, ENO3, MMP2, BUB1B, NCKAP1L, COL5A2, PKIA
**KICH**	SLC38A1, SEPT2, ARHGAP5, NPAS2, PLCB1, ATP6V1A, DAPK1, GNAI3, STAM2, RALB, LEPR, EPN2, ITPR2, HNF4A, CXCL12, COL4A1, CXCL14, F2RL1, ARRB1, CASP10
**KIRC**	NOTCH3, VAV1, GYS1, HSPA2, PFKFB4, COL4A2, IL12RB1, PDGFRB, FBP1, CORO1A, NCKAP1L, IL10RA, WAS, BTK, PTPN7, ABAT, ARHGEF1, COL1A2, LRDD, PTH1R
**KIRP**	PABPC1L, MDM4, DDC, PCK2, PDE2A, PTPRB, IQGAP2, ATP6V0D2, CACNA2D2, ATP1A3, SFRP1, FBP1, ASH1L, LRDD, PPP2R5D, MYH11, IGF2, LATS1, CCNB2, VAV1
**LIHC**	CCNB1, CDC20, PTTG1, CCNB2, PGF, GYS2, BUB1B, TTK, NKD1, COL6A3, CCND2, SGOL1, MRVI1, MRC2, NAT2, VIPR1, AXIN2, CACNA1C, WNT6, MMP1
**LUAD**	CDC20, CCNB1, JAM2, SGOL1, BUB1B, BIRC5, TTK, SCN7A, NCKAP1L, CNGA4, CDC25C, ESPL1, GRIN3B, BUB1, FIGF, PTPRB, MAD2L1, TEK, SLIT2, PTPRC
**LUSC**	BUB1, THOC4, TGFBR2, SGOL1, DPYSL2, SCN7A, TRPV2, CXCR2, PTPRB, CACNA2D2, CTSS, BTK, BIRC5, NCKAP1L, FCGR3B, COL6A3, WAS, CCNB2, AQP4, IL10RA
**PAAD**	CREB3L3, TUBAL3, BTK, NCKAP1L, IL10RA, WAS, IGF2, F5, CTNNA1, CORO1A, LATS1, CD19, CD48, ZAP70, PLA2G1B, CD79A, LILRB1, GRIN2C, HCLS1, PTPRC,
**PCPG**	MYH9, CXCL14, PFKL, HSPG2, NOTCH4, BUB1B, CCNB2, SEMA5B, NOTCH3, TAOK1, FZD10, LATS1, PDGFRB, CTSS, STAR, NOTCH1, CYP11A1, ITGA5, BIRC5, IQGAP2
**PRAD**	MYLK, MRVI1, FLNA, PPP1R12B, ADCY5, MYH11, CYSLTR2, ITGB6, FRMD6, HEPH, WNT9B, SORBS1, FZD7, CAMK2G, TCF7L1, DPYSL2, ACTN1, ATP2B4, EPHA2, SGCB
**READ**	PTK2, ATP6V1C1, LIFR, GNG2, ZAP70, MYLK, PPBP, CACNA2D1, MPDZ, TACR2, SLC4A4, LBP, PPP1R12B, IL10RA, DLG2, PDCD4, WAS, BTK, PDE5A, GNAO1,
**STAD**	CCNB1, MPDZ, BUB1B, MAD2L1, PLK4, MYLK, ORC1L, MYH11, CDC20, FLNA, SCN7A, CREB3L3, PARK2, CD86, ITGAL, COL6A3, CACNB2, BIRC5, CCNB2, TACR2
**THCA**	MET, KCNQ3, RUNX1, PTPRF, PDE5A, MAPK8IP3, ITGA3, FLNA, CCL2, FN1, IQGAP1, MYC, SDC4, BID, STAG1, SHANK2, PLA2R1, ITPR1, COL6A3, IL21R

Supplementary Table S4: Correlation between the 20 genes with the highest coreness values and Clinico-pathological characteristics of HCC

Variable	Associated genes
**Ajcc pathologic tumor stage**	GYS2, PLG, ADRA1A, HLA-A, MMP1, CXCL10
**AFP expression**	GYS2, PLG, ADRA1A, WNT10A, OR2A7, NKD1, FOXO1
**Vascular invasion**	GYS2, ADRA1A, AXIN2, NKD1
**Overall survival**	GYS2, MMP1, WNT10A
**Ishak fibrosis score**	GYS2, HLA-B, WNT10A, HLA-F

Supplementary Table S5: Correlation between the 20 influential genes identified by PageRank and Clinico-pathological characteristics of HCC

Variable	Associated genes
**Ajcc pathologic tumor stage**	SOCS2, GYS2, ETS2, ADRA1A, CYP2C8, ADH4, RCAN1
**AFP expression**	GHR, SOCS2, PCK1, GYS2, TSLP, ADRA1A, CYP2C8, ADH4, RCAN1
**Vascular invasion**	SOCS2, GYS2, ADRA1A, CYP2C8, ADH4, RCAN1
**Overall survival**	SOCS2, LIFR, GYS2, ADRA2B
**Ishak fibrosis score**	GYS2, MARCO
